# Nicotinamide mononucleotide ameliorates senescence in alveolar epithelial cells

**DOI:** 10.1002/mco2.62

**Published:** 2021-05-27

**Authors:** Tingting Fang, Jingyun Yang, Li Liu, Hengyi Xiao, Xiawei Wei

**Affiliations:** ^1^ Department of Cardiology West China Hospital Sichuan University Chengdu China; ^2^ State Key Laboratory of Biotherapy and Cancer Center West China Hospital Sichuan University and National Collaborative Innovation Center Chengdu China; ^3^ Lab of Aging Research and Nanotoxicology State Key Laboratory of Biotherapy and Cancer Center National Clinical Research Center for Geriatrics West China Hospital Sichuan University and National Collaborative Innovation Center Chengdu China

**Keywords:** aging, alveolar epithelial cells, NAD+, nicotinamide mononucleotide, senescence

## Abstract

Alveolar epithelial cells (ACEs) gradually senescent as aging, which is one of the main causes of respiratory defense and function decline. Investigating the mechanisms of ACE senescence is important for understanding how the human respiratory system works. NAD^+^ is reported to reduce during the aging process. Supplementing NAD^+^ intermediates can activate sirtuin deacylases (SIRT1–SIRT7), which regulates the benefits of exercise and dietary restriction, reduce the level of intracellular oxidative stress, and improve mitochondrial function, thereby reversing cell senescence. We showed that nicotinamide mononucleotide (NMN) could effectively mitigate age‐associated physiological decline in the lung of 8–10 months old C57BL/6 mice and bleomycin‐induced pulmonary fibrosis in young mice of 6–8 weeks. Besides, the treatment of primary ACEs with NMN can markedly ameliorate cell senescence phenotype in vitro. These findings to improve the respiratory system function and reduce the incidence and mortality from respiratory diseases in the elderly are of great significance.

## Graphical abstract

Supplementation of nicotin mononucleotide (NMN) or nicotinamide riboside (NR) can increase nicotinamide adenine dinucleotide (NAD+) synthesis, enhance SIRT1 activity, maintain mitochondrial function, protect nuclear DNA integrity, anti‐inflammatory and promote cellular antioxidant function, thereby resisting replicative and stressful induced senescence.

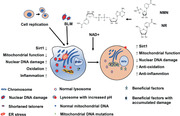

## 1 INTRODUCTION

One of the most significant changes in lung with aging is the decrease in the number and function of alveolar epithelial cells (ACEs).^1^, ^2^, ^3^ ACEs mainly locate at the terminal end of the respiratory tract and are the main components of the blood gas barrier. They are classified into type I alveolar epithelial cells (AECIs) and type II alveolar epithelial cells (AECIIs). AECIs account for 96% of the total AECs, while AECIIs only 4%. Interestingly, AECIIs in the lung are active progenitor and secretory cells, secrete alveolar surfactant, and maintain alveolar surface tension.^4^, ^5^ AECs constitute 99% of the surface area of the lung, playing a major role in lung functions: barrier, gas exchange, and immunomodulation. The senescence of these cells is the main reason for the decline of lung function in aging.^6^ Although AECs senescence is of great significance for the study of senescence‐related chronic lung diseases, its specific mechanism and role are still not fully understood.^7^, ^8^ NAD^+^ supplementation is reported to improve many aging‐related diseases, such as neurodegenerative diseases.^9^, ^10^, ^11^, ^12^ However, the effects of NAD^+^ precursors on pulmonary aging and AECs senescence have not been reported.

Recent studies have found that supplementation of NAD^+^ precursor nicotinamide mononucleotide (NMN) and nicotinamide riboside (NR) can increase the levels of NAD^+^ within cells.^10^, ^13^ Supplementation of NMN to AECs cultured in vitro can significantly alleviate the replication senescence of AECs, preventing age‐related physiological decline, increasing DNA repair function, and improving mitochondrial dysfunction and glucose intolerance.^13^, ^14^ Whether adding NAD^+^ precursor in vivo could alleviate the lung aging and improve ACEs senescence in vitro is still unclear.^15^

## 2 RESULTS

### 2.1 NMN relieves alveolar cell senescence in aged mice

**FIGURE 1:**
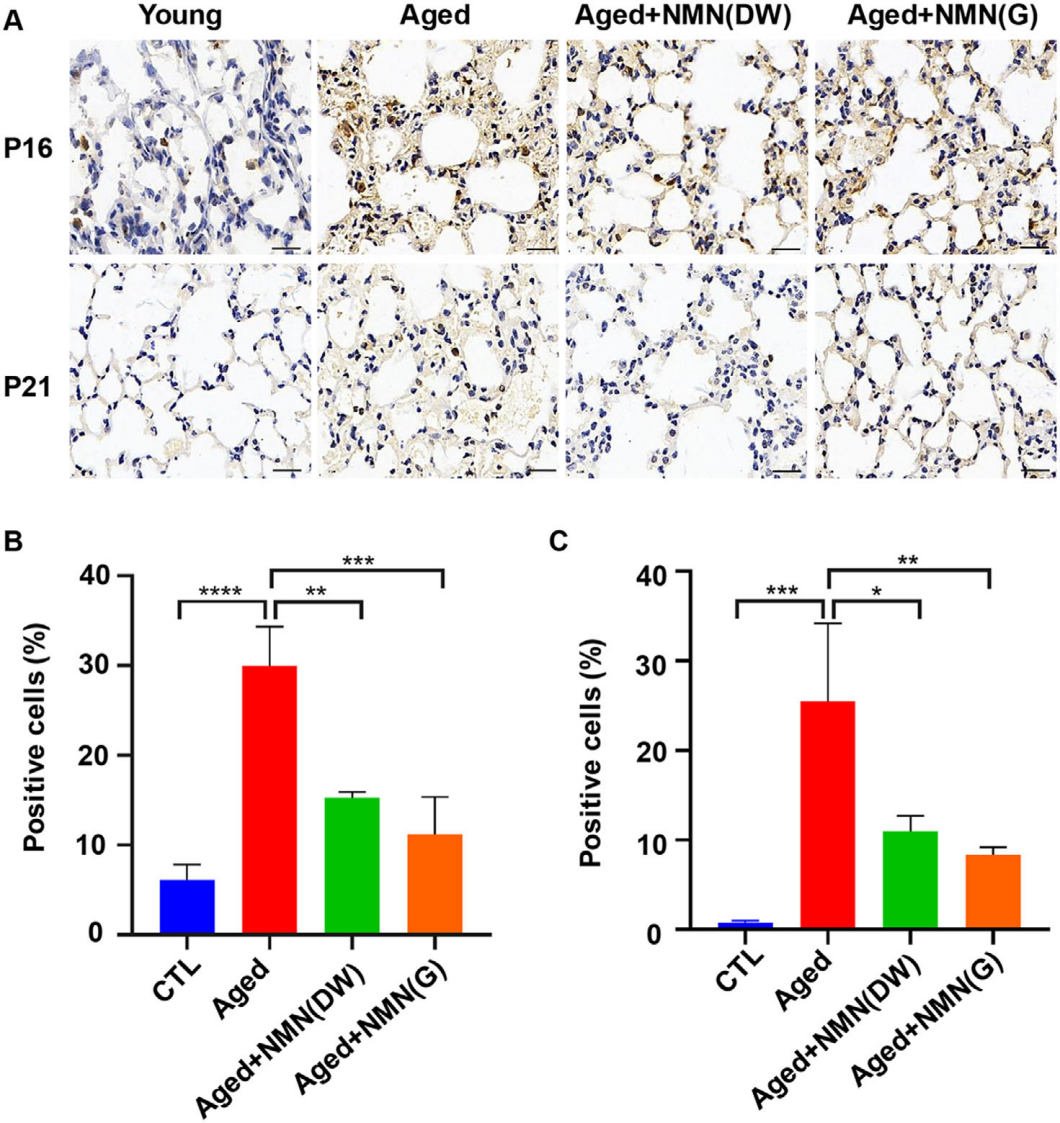
Age‐related proteins were significantly decreased in the lung tissues of NMN‐supplemented mice. (A) The P16,P21 (bar = 20 μm) immunohistochemical staining was performed on the paraffin sections of mice in the four groups (young control group, aged control group, aged and NMN group by drinking water (DW), and aged and NMN group by gavage (G), 10 mice in each group). (B) Five visual fields of mice lung tissue aging related protein P16 staining were randomly taken for statistical analysis. (C) Five visual fields of mice lung tissue aging related protein P21 staining were randomly taken for statistical analysis (*p < 0.05, **p < 0.01, ***p < 0.001, ****p < 0.0001)

The function of various tissues and organs of the body decreases with aging, accompanied by a decrease in intracellular NAD^+^, including the lungs.^13^, ^16^, ^17^, ^18^ However, whether the reduction of NAD^+^ mediates the senescence of alveolar cells and whether the supplementation of NMN could alleviate this phenotype had not been reported. We administered NMN to C57/BL6 mice aged 8–10 months at a dosage of 500 mg/kg/day for 2 months. After that, aging indexes of lung tissue were detected. Senescent cells accompanied by a reduced proliferative capacity, mainly due to the increase in expression of cell cycle inhibitor proteins.^19^ We found that aging‐related cyclic inhibitory proteins were significantly reduced in aging+NMN group by gavage, and aging+NMN group by drinking water compared with the aging group (Figure 1). Therefore, we believe the long‐term adding of NMN can be effective in improving lung aging.

### 2.2 NMN can relieve replicative senescence of ACEs

**FIGURE 2:**
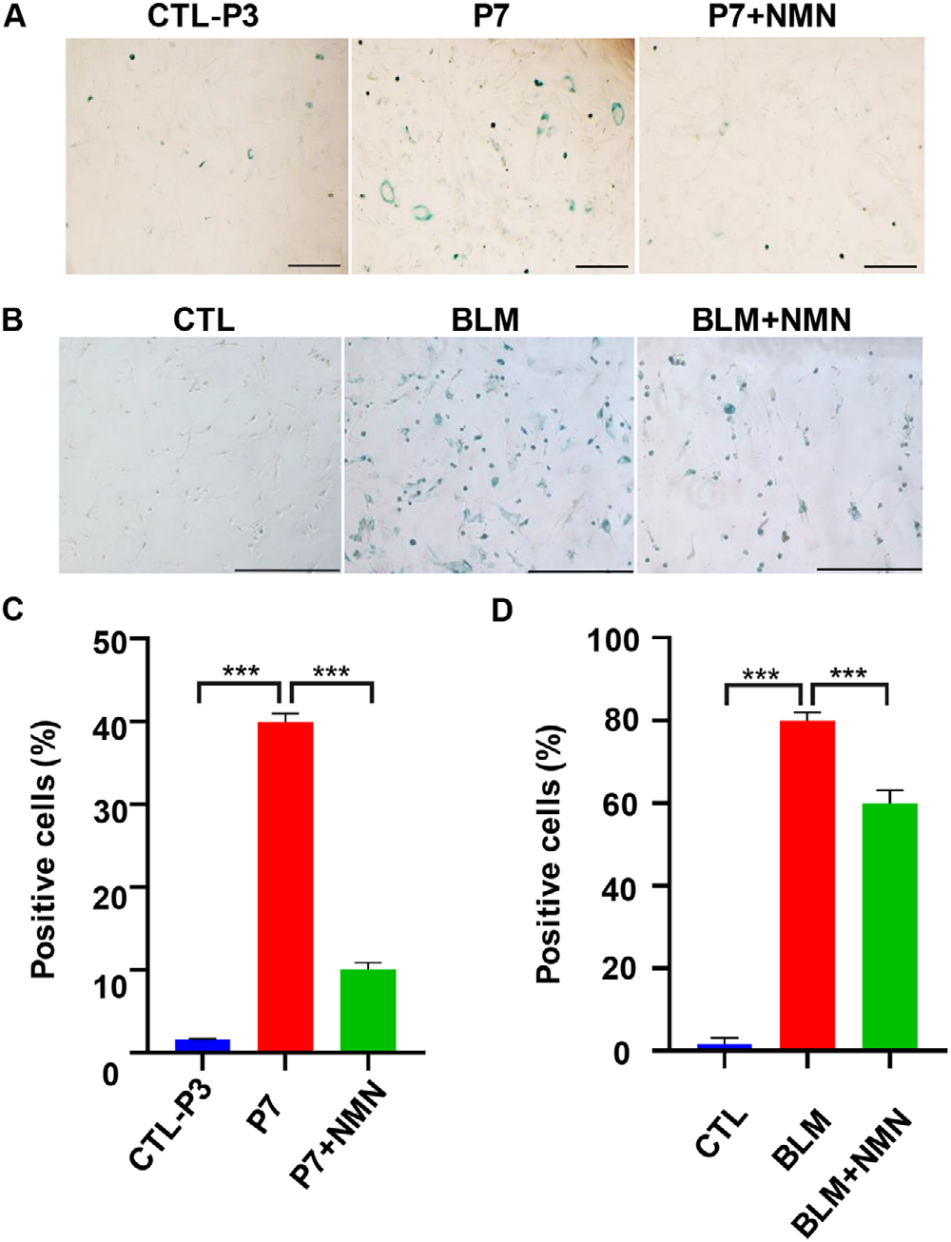
NMN improved cell senescence. (A) NMN mitigates alveolar epithelial cell senescence as shown by SA‐β‐gal staining under inverted microscope (scale bar = 20 μm); three sets of multiple holes were set in each group. (B) NMN mitigates bleomycin‐induced alveolar epithelial cell senescence as shown in SA‐β‐gal staining under inverted microscope (scale bar = 500 μm). (C) Five fields of alveolar epithelial cell senescence SA‐β‐gal staining were randomlycalculated for statistical analysis. (D) Five visual fields of bleomycin‐induced alveolar epithelial cell senescence SA‐β‐gal staining were randomly calculated for statistical analysis (****p < 0).

On the basis of animal experiments, we wanted to further explore whether NMN could directly alleviate ACE replicative senescence and stress‐induced senescence. In order to confirm this hypothesis, we isolated primary ACEs. Cells appeared senescence phenotype when they were passaged to 7th/8th generation. The size of the cells appeared to be rounded, significantly larger in morphology and cell proliferation was inhibited. One of the reasons for cell senescence is the decrease of NAD^+^, the imbalance of oxidation/antioxidant system, and the stress‐induced senescence caused by increased oxidative stress.^20^, ^21^, ^22^ NMN, the precursor of NAD^+^, can effectively increase the level of NAD^+^ in vivo.^14^ We found that NMN supplementation could effectively alleviate the senescence of ACEs. NMN was added to the cells from passage 4th to passage 7th (P7). And the phenotype of cell senescence was significantly improved compared with the control group (P3). Senescence‐related SA‐β‐gal staining was found to be significantly reduced in ACEs in the 7th/8th passages (Figure 2A and C). In conclusion, the addition of NMN could effectively alleviate the replication of lung primary ACEs.

### 2.3 NMN alleviated bleomycin‐induced alveolar cell senescence in mice

**FIGURE 3:**
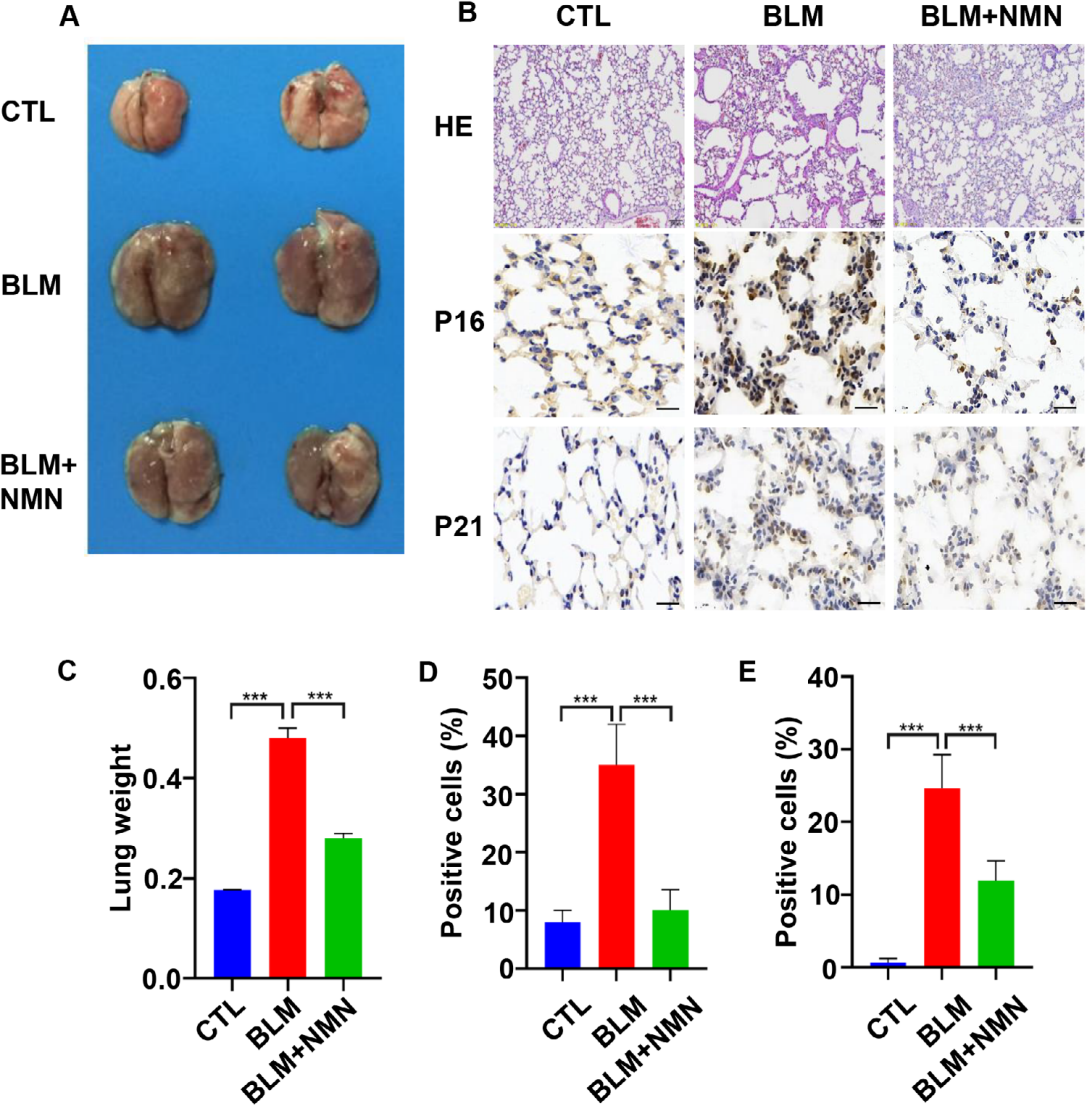
NMN improved the appearance and weight of lung. (A) Appearance of mice lung tissue anatomy. (B) HE staining (scale bar = 100 μm) of mice lung tissue, and P16 (scale bar = 20 μm), P21 (scale bar = 20 μm) immunohistochemical staining were performed on paraffin lung sections of mice treated with bleomycin. (C) The weight of lung tissue in mice. (D) Five visual fields of P16 immunohistochemical staining were randomly taken for statistical analysis. (E) Five visual fields of P21 immunohistochemical staining were randomly taken for statistical analysis (**p < 0.01, ***p < 0.001, ****p < 0.0001)

**FIGURE 4:**
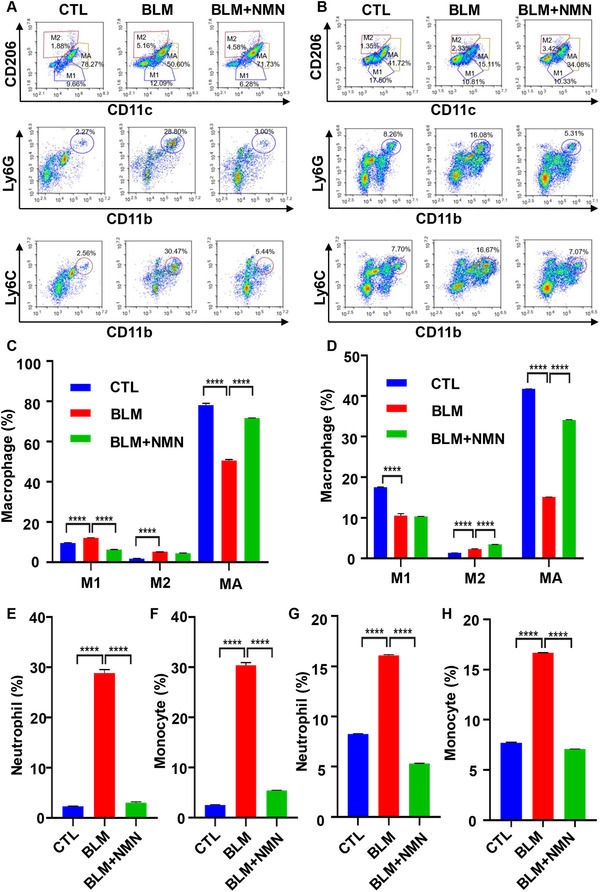
NMN improved pulmonary inflammation. (A) NMN alleviated bleomycin‐induced pulmonary inflammation in mice. The infiltration of macrophages, neutrophils, and monocytes in lung tissue was detected by flow cytometry. (B) Flow cytometry was used to detect macrophages in alveolar lavage fluid, three mice in each group. Macrophage activation can be divided into classically activated M1 macrophage (classically activated macrophage) and selectively activated M2 macrophage (alternatively activated macrophage). (C) Macrophages in lung tissue were detected by flow cytometry, three mice in each group. AM are alveolar macrophages, which play a demonstrative role during the pathogenesis of lung injury. In the BLM model group, mouse alveolar macrophages were damaged and the percentage decreased. After the administration of NMN, the damage was significantly improved, resulting in a recovery of its percentage. M1 type macrophages are generally activated by interferon‐γ and bacterial lipopolysaccharide (LPS). M1 mainly secretes proinflammatory factors and plays an important role in the early stages of inflammation. M2 type macrophages are activated by Th‐2 cytokines, such as IL‐4, IL‐13, and immune complexes. M2 expression inhibits inflammatory factors and plays a role in inhibiting inflammation and tissue repair. (D) Macrophages in alveolar lavage fluid were detected by flow cytometry, three mice in each group. (E) Neutrophils in lung tissues were detected by flow cytometry, with three mice in each group. (F) Flow cytometry was used to detect monocyte in lung tissues, three mice in each group. (G) Neutrophils in alveolar lavage fluid were detected by flow cytometry, with three rats in each group. (H) Flow cytometry was used to detect monocyte in alveolar lavage fluid, three mice in each group. (****p < 0.0001)

NMN can alleviate the replicative senescence of alveolar cells with aging. Whether it could alleviate the stress‐induced alveolar aging needed further study. Bleomycin (BLM) is a member of the glycopeptide antibiotic family and has an effective antitumor activity. Its main toxic side effect is pulmonary fibrosis, and the mechanism is genomic instability and ROS production.^23^ BLM‐induced pulmonary fibrosis is based on the induction of ACE senescence, thereby damaging the regeneration of ACEs.^24^ The study found that BLM induced cell senescence accompanying with pulmonary fibrosis, by inducing cell apoptosis. Therefore, we used BLM to induce pulmonary primary epithelial cell senescence, attempting to detect whether NMN could relieve the senescence of ACEs. It has been found that BLM dose is 2 mg/kg, on the 21st day of treatment. So, we gave BLM to mice by cough method and began NMN intragastric administration at a dose of 500 mg/kg/day. After 21 days of gavage, senescence‐related indicators were detected. It was found that the appearance of the lung in NMN group was significantly improved, and the lung weight was significantly decreased (Figure 3A and B). Through HE staining of mice lung tissue, we found that NMN can alleviate the destruction of alveoli and the inflammatory infiltration of the interstitial caused by BLM (Figure 3C). Besides, we performed flow cytometry of lung tissue and alveolar lavage fluid and found that the inflammatory cells in the NMN‐added group were significantly reduced (Figure 4). Immunohistochemical staining showed that the expression of senescence‐related cycle inhibitor protein decreased significantly in NMN‐supplemented group (Figure 3C–E). Therefore, we believe that the addition of NMN can significantly improve BLM‐induced ACE senescence.

### 2.4 NMN alleviated BLM‐induced ACE senescence

Similarly, based on the in vivo model of BLM induced alveolar cell senescence, we detected whether NMN could effectively alleviate the senescence of ACEs. We found that NMN could effectively alleviate BLM‐induced senescence. The dosage of BLM was 5 μg/ml. Induction time was 3 days, and then 3 days later. BLM‐treated cells added NMN (500 μm/ml) at the same time. Activity of SA‐β‐gal in BLM‐induced ACEs was significantly lower in NMN‐added group than that in nonadded group (Figure 2B and D). In conclusion, the addition of NMN can effectively alleviate BLM‐induced lung primary cell senescence.

## 3 DISCUSSION

We tried to understand whether NMN could alleviate the replication and stress‐induced senescence in human ACEs via mice models. We found that both in vivo and in vitro, ACEs of the same passage number were significantly reduced in NMN supplemented and nonsupplemented groups. The decreased numbers of cells were not changed by the BLM treatment nor the BLM treatment with NMN addition, senescent ACEs were also significantly reduced. We also found that NMN reduced BLM‐induced inflammation in mouse lungs. Our data suggested that NMN could effectively alleviate the replicative and stress‐induced senescence of ACEs in vivo and in vitro. It provides a preventive and therapeutic approach for aging‐related chronic lung diseases and lung injury caused by external stimuli in the future.

**FIGURE 5:**
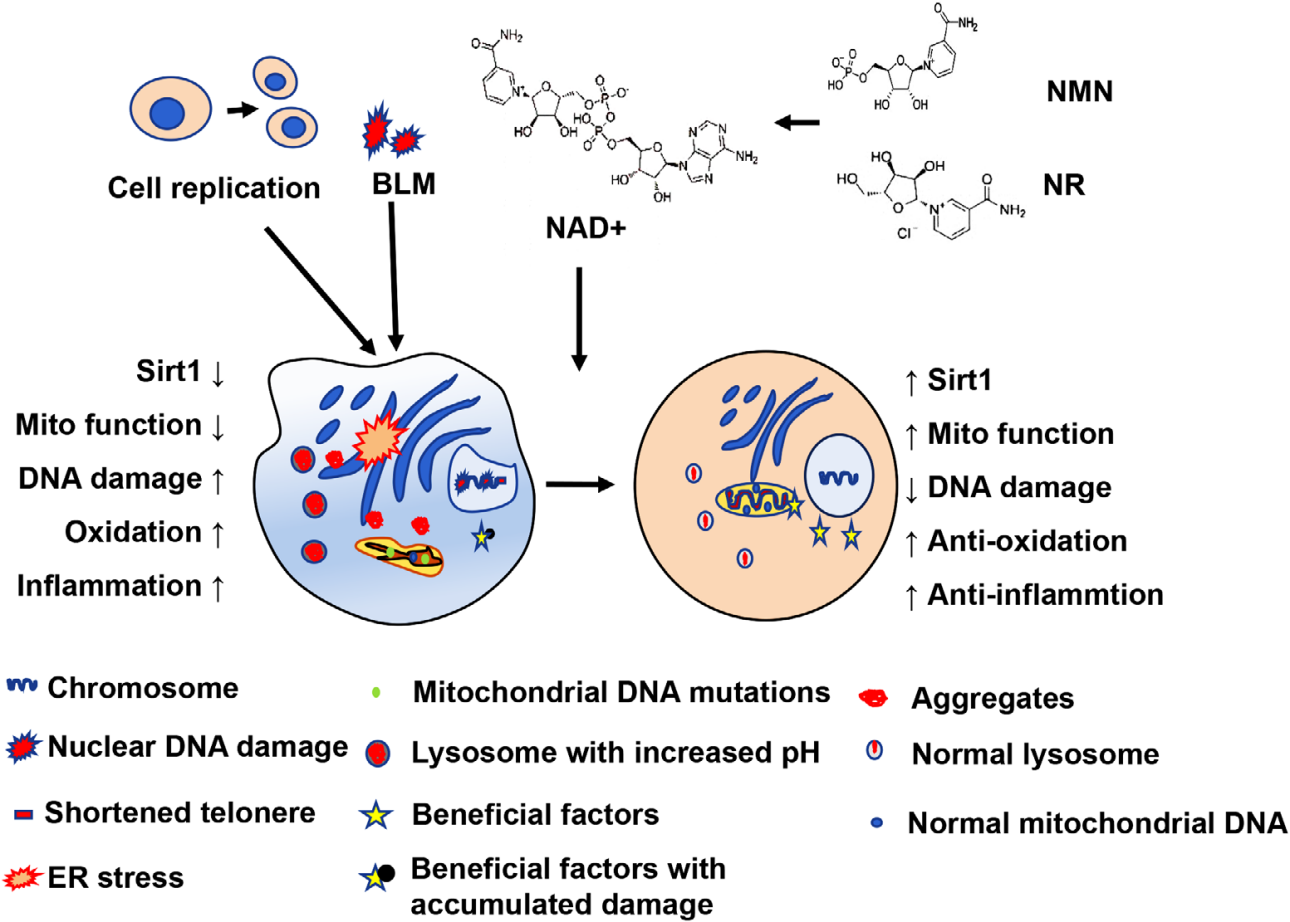
The pathways of NMN complement to against cellular aging. Supplementation of NMN or NR can increase NAD^+^ synthesis, enhance Sirt1 activity, maintain mitochondrial function, protect nuclear DNA integrity, anti‐inflammatory, and promote cellular antioxidant function, thereby resisting replicative and stressful‐induced senescence

NAD^+^ decreases in vivo with aging and is considered to be an important regulator of age‐dependent pathological processes.^25^ NAD^+^ participates in various physiological activities in the body and is a key cofactor for glycolysis, tricarboxylic acid cycle, and oxidative phosphorylation, also in various redox reactions in cells.^26^, ^27^, ^28^, ^29^, ^30^, ^31^ The antiaging effect of NAD^+^ supplementation has been confirmed in many tissues and organs, such as satellite cells, angiogenesis, and myocardium of skeletal muscle.^13^ NMN and NR can be used as a nutritional additive to increase the level of NAD^+^ in vivo, thereby preventing the occurrence of aging (Figure 5).^32^, ^33^, ^34^, ^35^

There are many factors leading to cell senescence, including oxidative stress, mitochondrial dysfunction, and so on.^36^ Studies have shown that the toxicity of BLM in cells is mainly due to genetic instability and increased oxidative stress.^37^ NMN supplementation not only decreases the level of oxidative stress in cells, but also reduces the damage of cellular function induced by oxidative stress, including genetic instability, improves mitochondrial functions, and increases the activity of NAD^+^‐dependent enzymes (e.g., SIRT family).^38^ Aging is a complex process; we have not studied in depth how NMN prevented the aging of ACEs. Cell senescence is a complex process accompanied by changes in gene expression and secretion of cytokines and proteases (SASP)^39^, ^40^, ^41^, ^42^; however, we did not perform SASP test in this experiment. Current studies have shown that senescent ACEs had a high level of proinflammatory cytokine phenotype.^25^ However, this age‐related phenotype was not detected in this study. Although our data did not exclude other lung parenchyma cells or extra‐pulmonary factors, nor separating alveolar type I and type II cells. However, our study was based on cells in the lungs, so there is no doubt that addition of NMN to alveolar cells decreases senescence cells absolutely.

In conclusion, we found that the long‐term addition of NMN could effectively improve ACE replication and stress‐induced senescence in vivo and in vitro. Dietary supplementation of NMN might be a new and effective way to prevent and reduce aging‐related lung diseases and stress‐induced lung injury in the future.

## 4 MATERIALS AND METHODS

### 4.1 Animals

Unless otherwise stated, all biochemical reagents used in this study were purchased from Sigma Chemicals (St. Louis, Missouri, MO, USA). Antibodies listed for mouse samples were of commercial grades and validated by manufacturers based on their data sheets. All animal experiments were performed according to the Guidelines of the Institutional Animal Care and Use Committee of Sichuan University (Chengdu, Sichuan, China), and the protocols were approved by the Institutional Animal Care and Use Committee of Sichuan University.

### 4.2 Animal treatment

SPF grade C57/BL6 mice (female, 8–10 months or 6–8 weeks old, 28–30 g or 19–21 g) were purchased from Beijing Weitong Lihua Laboratory Animal Technology Co. Ltd. and raised in SPF grade animal room.

### 4.3 Mice were administered with gavage

Young (6–8 weeks) and aged (8–10 months) mice were used. The mice were divided into young control group, aged control group, aged and NMN group by drinking water, and aged and gastric NMN group (10 mice in each group). The water intake was determined in advance to calculate the concentration of NMN in drinking water. Mice were given NMN of 500 mg/kg/day for 8 weeks. Mice in BLM‐induced aging model were divided into control group, BLM‐induced aging group, and BLM‐induced aging NMN additional group. Mice were given 500 mg/kg NMN by intragastric administration everyday. BLM was given 2 mg/kg by lingual and laryngeal drip. The mice were then given the same dose of NMN by intragastric administration every day for 3 weeks.

### 4.4 Cell culture and treatment

Primary lung epithelial cells were isolated from C57/BL6 mice lung (6–8 weeks), cultured with RPMI 1640 (Gibico) and 10% fetal bovine serum supplemented with 100U penicillin and streptomycin. Mice were sacrificed and the lung tissues were removed. Lung tissues were washed with saline and minced into very fine pieces with ophthalmic scissors followed by adding type IV collagenase. Tissues were digested at 37°C for 1 h. After the filtration with a mesh, the digested cells were isolated with centrifugation (800 rpm/3 min) and the supernatant was discarded. Next, erythrocyte lysis buffer, mixed were added for 3 min, followed by the centrifugation (1200 rpm/3 min) to discard the supernatant. The isolated cells were resuspended and the medium was changed the next day. Cells were grown in a humidified atmosphere at 37°C with 5% CO_2_. When the cell grows to about 60%, it is treated with 5 μg/ml of BLM. Cells were grown in a humidified atmosphere at 37°C with 5% CO_2_. The cells are passaged once every 3 days.

### 4.5 SA‐β‐gal staining

SA–β‐gal activity was quantitatively measured by Senescence β‐Galactosidase Staining Kit (Beyotime). Primary lung epithelial cells cultured in 24 well plates were washed with PBS or HBSS twice before adding 1 ml of beta galactosidase staining fixative. Then, fixed at room temperature for 15 min. Gettering cell fixation, cells were washed with HBSS or PBS three times for 3 min each. Then, the cells were fixed at room temperature for 15 min. In the next step, cells were washed with HBSS or PBS for three times. The cells were sealed with paraffin and were incubated overnight at 37°C. Photographs were taken under a standing fluorescence microscope. Photographs were taken under a standing fluorescence microscope.

### 4.6 Immunohistochemical staining

The deparaffinized and rehydrated lung sections were exposed to 3% H_2_O_2_ in methanol for 30 min to quench endogenous peroxidase activity after antigen retrieval using the citrate buffer (0.01 M, pH 6.0). Nonspecific binding of antibodies to the tissue sections was blocked by incubating sections with 5% normal goat serum in PBS for 30 min. Lung tissue sections were incubated with primary p16 (Abcam; 1:100) or p21 (Abcam; 1:1000) antibody and p53 (Servicebio; 1:200) at a titer overnight at 4°C. After being washed, sections were incubated with secondary antibody biotinylated HRP goat anti‐rabbit IgG (servicebio) for 1 h, and DAB was used as peroxidase substrate. The counterstaining with hematoxylin was then performed before examination under a light microscope.

### 4.7 Flowcytometry

Single cell suspension of the lung tissues was stained with antibodies against CD45 conjugated with Percpcy 5.5, CD11B conjugated with APC; LY6G conjugated with PE; LY6C conjugated with FITC, F4/80 conjugated with PE; CD206 conjugated with APC. Finally, the cells were resuspended with 200 μl PBS and analyzed with flow cytometry.

### 4.8 Statistical analysis

Data were analyzed by a two‐tailed Student's *t* test and ANOVA. Statistical analysis was performed using GraphPad Prism 6.

## CONFLICT OF INTERESTS

The authors have no conflicts of interest to disclose.

## ETHICS APPROVAL

All studies on animals were performed after approval by the Ethics Committee of Sichuan University, in compliance with Guidelines for the Use and Care of Small Laboratory Animals.

## AUTHOR CONTRIBUTIONS

Xiawei Wei and Hengyi Xiao provided study concepts and designed the study. Tingting Fang, Jingyun Yang, and Li Liu did the experiments. Tingting Fang was involved with data acquisition. Tingting Fang, Jingyun Yang, and Li Liu were involved with quality control of data and algorithms. Tingting Fang, Jingyun Yang, and Xiawei Wei edited the manuscript. Xiawei Wei and Hengyi Xiao reviewed the manuscript.

## Data Availability

All data included in this study are available upon request by contact with the corresponding author.
